# A Scoping Review of Preferences of Men Who Experienced Sexual Assault: Implications for Adaptation of Trauma-Focused Cognitive Behavioral Therapies

**DOI:** 10.1177/15579883241260512

**Published:** 2025-02-10

**Authors:** Lydia Gamache, Laurence Dubé, Geneviève Belleville

**Affiliations:** 1 School of psychology, Université Laval, Québec City, Québec, Canada

**Keywords:** male sexual assault, trauma, psychological services, scoping review

## Abstract

About 1 in 10 men experiences sexual assault, resulting in various difficulties most frequently associated with post-traumatic stress disorder. However, trauma-focused cognitive behavioral therapies (TF-CBT) seem less effective for men who experienced sexual assault compared to women. Efficacy of TF-CBT could be improved by adapting interventions according to the empirical data detailing men’s preferences regarding psychological services. This scoping review aimed to document preferences of men who experienced sexual assault regarding psychological services, and to explore barriers and motivators to help-seeking for this population. A systematic approach was used to gather literature describing preferences regarding psychological services, and barriers and motivators to help-seeking. Thirty-five peer-reviewed studies and two non-peer reviewed reports met inclusion criteria. Data from included articles were extracted using a systematic extraction grid. A thematic content analysis was conducted to synthesize and present the results from the 37 studies. The number of empirical studies on preferences regarding psychological services was limited as only five documented preferences, all related to the clinician’s characteristics (e.g., clinician’s gender) and the type of intervention (e.g., action-oriented). Most studies reviewed barriers to help-seeking. The barriers most frequently identified were adherence to masculine norms and to myths about male sexual assault. Injury or substance abuse following sexual assault often act as motivators to help-seeking for men. To adapt TF-CBT to men who experienced sexual assault, researchers and clinicians should accommodate and further study these men’s preferences, consider their motivators regarding help-seeking and alleviate barriers to help-seeking, notably by deconstructing masculine norms.

Sexual assault, defined as a sexual conduct perpetrated without consent, such as an unwanted sexual intercourse, sexual touching, or kissing, is a public health issue affecting the health and well-being of millions of people worldwide (S. Khan et al., 2020). Although women report having experienced sexual assault more often than men, a growing body of literature provides evidence on the prevalence and consequences of having experienced sexual assault among men (for a review, see Peterson et al., 2011). In North America, about 1 in 10 men will experience sexual assault in their lifetime (Tourigny et al., 2008) and face physical and psychological consequences, such as the development of anxiety, depression or, more frequently, post-traumatic stress disorder (PTSD; Heidt et al., 2005; Morris et al., 2014; Tewksbury, 2007).

Although studies have identified that male victims of sexual assault experience PTSD symptoms as severe as female victims (Elliott et al., 2004; Guina et al., 2019), there is also evidence of consequences to sexual assault that are more common among men than women. Notably, in a national study comparing the symptomatology of both genders who had experienced sexual assault, Elliott and colleagues (2004) reported that men had higher levels of sexual concerns and dysfunctional sexual behaviors than women and engaged more often in self-destructive or health-damaging behaviors (e.g., unsafe sexuality, smoking, substance abuse).

Psychological interventions have proven effective in reducing PTSD symptoms and psychological distress for victims of sexual assault, regardless of gender. According to expert consensus guidelines, trauma-focused cognitive behavioral therapies (TF-CBT) represent the gold standard for the treatment of PTSD (e.g., Bisson et al., 2013; Forman-Hoffman et al., 2018). More specifically, prolonged exposure therapy (PE), which consists of exposing a patient to reminders of the traumatic event to promote the emotional and cognitive processing of the trauma, has demonstrated effectiveness in reducing PTSD symptoms and improving the quality of life of sexual assault victims (Cusack et al., 2016; Ehring et al., 2014). Cognitive processing therapy (CPT), which targets trauma-related dysfunctional beliefs, has also been validated among victims of sexual assault. Effectiveness studies have reported significant reduction in PTSD and depression symptoms for both genders (Chard, 2005; Resick & Schnicke, 1992; Schnurr et al., 2022; see Schnyder and Cloitre [2022] for further details on TF-CBT). Eye Movement Desensitization Reprocessing (EMDR), which involves desensitizing an individual to trauma using a series of eye movements, is another TF-CBT that appears effective in reducing PTSD symptoms, but its efficacy in early interventions for PTSD has yet to be fully established (Covers et al., 2021).

Studies have demonstrated that only a small proportion of men seek psychological help (AL-Asadi, 2021; Gallegos et al., 2015; Masho & Alvanzo, 2010). As of 2021 in Canada, for every seven women who sought psychological services, only one man did likewise (AL-Asadi, 2021). For men who do seek help, TF-CBT appears to be effective, but to a lesser extent than for women. Felmingham and Bryant’s (2012) randomized controlled trial reported a similar significant reduction in PTSD symptoms between male and female victims of sexual assault immediately after attending PE or CPT combined with PE. However, reduced maintenance of treatment gains was observed at 6-month follow-up for men who completed PE only compared to women. Another study investigated the impact of gender on response to PE and CPT (A. J. Khan et al., 2020). Following CPT, women demonstrated a significantly greater reduction in PTSD symptom severity than men, whereas no gender difference in treatment response was observed after PE. In addition, Békés and colleagues (2016) have reported greater secondary gains (e.g., quality of life, support-seeking, supportive interactions) in women attending a TF-CBT for PTSD compared to men. However, no statistically significant gender difference was observed on PTSD symptoms.

A way to improve the efficacy of TF-CBT for male victims of sexual assault could be to adapt interventions according to their preferences. Indeed, a meta-analysis including 53 studies and more than 16,000 clients reported that preference accommodation is related to better treatment outcomes (Swift et al., 2018). To achieve adequate treatment adaptations for men who experienced sexual assault, there is a need to better understand their preferences regarding psychological services. Therefore, a scoping review was conducted to document the preferences regarding psychological services identified in the literature for men who experienced sexual assault. Other aspects that may influence the uptake of psychological services by men who experienced sexual assault are barriers and motivators to help-seeking. Thus, the secondary objective of this scoping review was to explore the specific barriers and motivators to help-seeking among men who experienced sexual assault.

## Method

The Ethics Committee of the Institution in which the research was conducted waived the need for ethical approval for this research project as it was based exclusively on the secondary use of published anonymous information. Preferences regarding psychological services being dispersed across multiple disciplines and methodologies, a scoping review approach, which aims to map available literature in a number of fields (Munn et al., 2022) and allow for the inclusion of multiple types of evidence, was chosen. A scoping review of the literature was thus performed to identify empirical studies documenting preferences regarding psychological services, as well as barriers and motivators to help-seeking, in men who experienced sexual assault. Preferences regarding psychological services were defined as options that are considered most appropriate by an individual under given circumstances. Psychological services included any psychological help provided by a trained provider in a variety of settings (e.g., psychotherapy, crisis, or community intervention) or modalities (e.g., face-to-face, telephone, video conferencing, online platform). Barriers to help-seeking referred to reasons reported by men who experienced sexual assault for not wanting or being able to obtain psychological services. Motivators to help-seeking were operationalized as elements identified as causing or encouraging a person to consult services.

### Search Strategy

A systematic approach based on the extension for scoping reviews of the Preferred Reporting Items for Systematic Reviews and Meta-Analyses (PRISMA-ScR; Tricco et al., 2018) and the recommendations of the Joanna Briggs Institute (JBI) scoping review methodology group (Munn et al., 2022) was used to search for relevant literature. To gain a multidisciplinary view of service preferences, three databases (PsycNet, PubMed and Eric) were searched. In total, 23 search equations (see Appendix) were created based on keywords related to the topic of interest. Keywords addressed men and gender differences (i.e., “men,” “male,” “women,” “gender,” “sex,” and “differences”), psychological treatments (i.e., “treatment,” “intervention,” “therapy,” “efficacy,” and “cognitive behavioral”), psychological treatment preferences (i.e., “preferences”), traumatic events (i.e., “PTSD,” “sexual assault,” and “sexual abuse”), and others (i.e., “symptoms” and “experiences”). When a search equation yielded more than 1,000 results, an advanced search was performed using the “Keywords” filter on PsycNet. “MeSH terms” on PubMed and “Descriptor” on Eric were added to each keyword in the equation. Searches were conducted in May 2021.

### Screening Process

**Figure 1.**
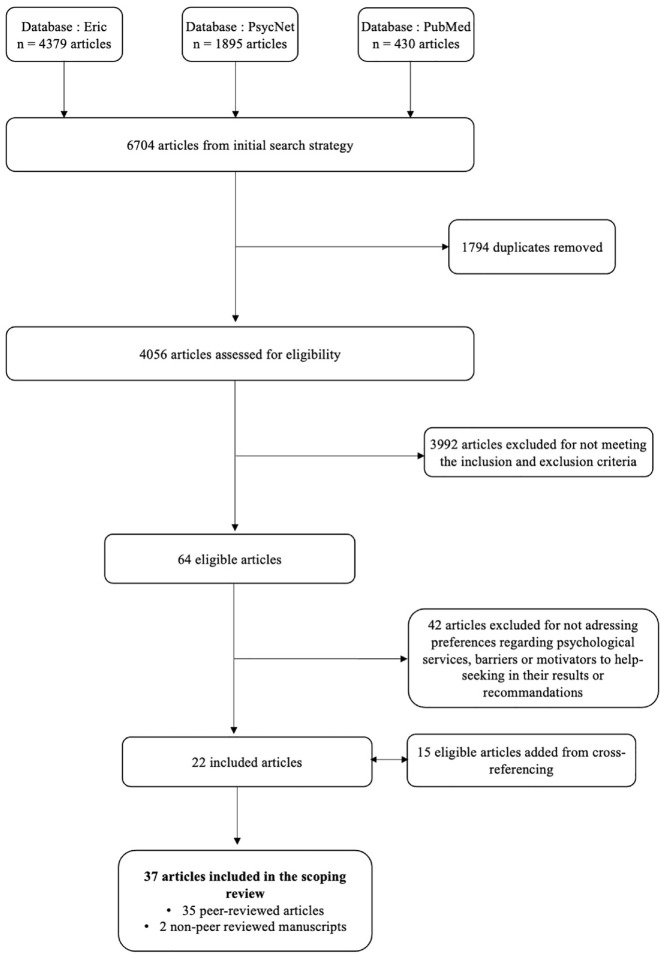
Flowchart of the Selection of Articles

Figure 1 presents the outcomes of the data selection process. Initial searches identified 4,056 articles, which were reviewed for eligibility based on the title and abstract. The articles had to be published over a 15-year span, between 2006 (a year marked by a notable surge in studies on male sexual assault indexed on PubMed) and May 2021 (year when the search was conducted), address sexual violence and men or gender differences. Peer-reviewed articles, theses, and dissertations, and government reports and non-peer reviewed commentary articles were included to widen the search. Reviews were included as they provided valuable recommendations and insights that complemented the primary research findings. Only case studies were excluded given their limited generalizability. Articles that targeted juvenile population were also excluded. The eligibility of the studies was assessed by two trained graduate students. Disagreements were resolved by discussion, until full inter-rater agreement was achieved. A total of 64 articles were eligible based on title and abstract.

Eligible full articles had to specifically address preferences regarding psychological services, barriers to help-seeking, or motivators to help-seeking in their results or recommendations. Forty-two studies were excluded on this basis after reading the articles. Data from 22 articles were extracted by the first two authors with a systematic extraction grid detailing sample characteristics, study design, objectives, and hypotheses, and relevant results and discussion elements. Inter-rater agreement was realized. Disagreements were resolved by discussion. Selected articles were cross-referenced, resulting in 15 additional articles. A total of 37 articles, of which 35 are peer-reviewed and two are non-peer reviewed reports, were included in the scoping review. See Table 1 for a description of the studies.

**Table 1.** Description of Included Studies (N = 37)

Literature reviews (*n* = 9)					
References	Year	N_articles_	Context of the assault		Peer-reviewed
Allard et al.	2011	74	Military sexual trauma		Yes
Fogler et al.	2008	NR	Childhood sexual abuse (clergy)		Yes
Hooper & Warwick	2006	NR	Childhood sexual abuse		Yes
Hoyt et al.	2011	29	Military sexual trauma		Yes
Morris et al.	2014	NR	Military and adult sexual assault		Yes
O’Brien et al.	2015	NR	Military sexual trauma		Yes
Peterson et al.	2011	87	Adult sexual assault		Yes
Turchik et al.	2016	NR	Adult sexual assault/rape		Yes
Turchik & Edwards	2012	NR	Adult sexual assault/rape		Yes
Randomized controlled trial (*n* = 1)					
References	Year	N_participants_	Population	Design	Peer-reviewed
Turchik et al.	2014	153 (male only)	Military sexual assault	Cross-sectional mixed	Yes
Treatment studies (*n*= 3)					
Hoyt et al.	2012	NR	Military sexual trauma	Longitudinal quantitative	Yes
O’Brien et al.	2008	175 (83% female)	Military sexual trauma	Longitudinal quantitative	Yes
Voelkel et al.	2015	481 (56.8% male)	Military sexual trauma	Longitudinal quantitative	Yes
Cross-sectional studies (*n* = 10)					
Light & Monk-Turner	2009	219 (male only)	Adult sexual assault/rape	Cross-sectional quantitative	Yes
Monk-Turner & Light	2010	219 (male only)	Adult sexual assault/rape	Cross-sectional quantitative	Yes
Pavao et al.	2013	118,498 (99.3% male)	Military sexual trauma	Cross-sectional quantitative	Yes
Sable et al.	2006	215 (54.7% female)	Male sexual assault	Cross-sectional mixed	Yes
Schry et al.	2015	2,546 (81.6% male)	Military sexual trauma	Cross-sectional quantitative	Yes
Snipes et al.	2017	782 (68.3% female)	Adult sexual assault/rape	Cross-sectional quantitative	Yes
Turchik	2012	4,458 (74.9% female)	Military sexual trauma	Cross-sectional quantitative	Yes
Turchik et al.	2012	302 (male only)	Military sexual trauma	Cross-sectional quantitative	Yes
Voller et al.	2015	1,827 (male only)	Adult sexual assault/rape	Cross-sectional quantitative	Yes
Wolfe et al.	2006	76 (male only)	Childhood sexual abuse (clergy)	Cross-sectional quantitative	Yes
Cohort study (*n* = 1)					
A. J. Khan et al.	2020	9,711 (88.2% male)	Military sexual trauma	Longitudinal quantitative	Yes
Case-control studies (*n* = 2)					
O’Leary & Gould	2009	1,378 (male only)	Childhood sexual abuse	Cross-sectional mixed	Yes
O’Leary	2009	1,378 (male only)	Childhood sexual abuse	Cross-sectional quantitative	Yes
Qualitative studies (*n* = 9)					
Alaggia & Millington	2008	14 (male only)	Childhood sexual abuse	Cross-sectional qualitative	Yes
Dorahy & Clearwater	2012	9 (male only)	Childhood sexual abuse (clergy)	Cross-sectional qualitative	Yes
Elder et al.	2017	21 (male only)	Military sexual trauma	Cross-sectional qualitative	Yes
Hovey et al.	2011	49 (male only)	Childhood sexual abuse	Cross-sectional qualitative	Yes
Isely et al.	2008	9 (male only)	Childhood sexual abuse (clergy)	Cross-sectional qualitative	Yes
Kia-Keating et al.	2010	16 (male only)	Childhood sexual abuse	Cross-sectional qualitative	Yes
Sorsoli et al.	2008	16 (male only)	Childhood sexual abuse	Cross-sectional qualitative	Yes
Teram et al.	2006	95 (51.5% male)	Childhood sexual abuse	Cross-sectional qualitative	Yes
Turchik et al.	2013	20 (male only)	Military sexual trauma	Cross-sectional qualitative	Yes
Gray literature (*n* = 2)					
References	Year	Type of document	Population	Type of results	Peer-reviewed
Kakhnovets & Holonan	2007	Clinical review	Military sexual trauma	Qualitative	No
Steiger et al.	2010	Research report	Military sexual trauma	Quantitative	No

*Note.* NR = not reported.

### Data Synthesis

To guide the presentation of the results, a thematic analysis was conducted with the main findings and recommendations of the studies. The analysis followed an iterative process, as a codebook was created and then refined as the thematic analysis progressed, using the NVivo software. The first two authors of the article conducted the analysis separately, which resulted in an excellent inter-judge agreement (K= 0.94), and then reached consensus on disagreements through discussion.

## Results

### Overview of Identified Literature

**Figure 2.**
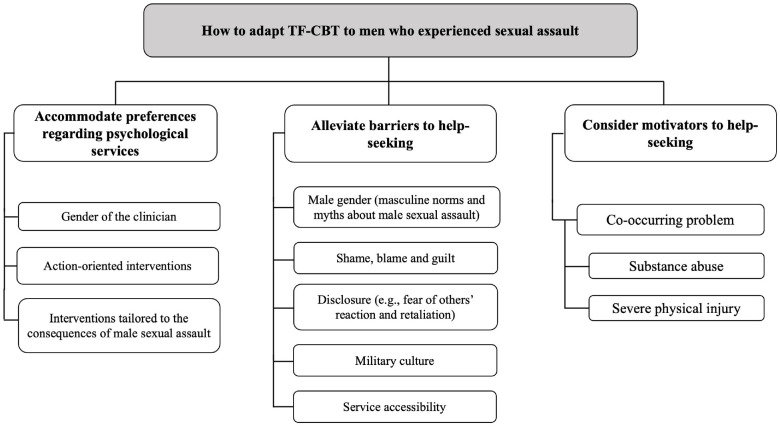
Overview of Reviewed Findings on Adaptations of TF-CBT to Men Who Experienced Sexual Assault

This scoping review collated literature on preferences regarding psychological services, as well as barriers and motivators to help-seeking from 35 peer-reviewed articles and two non-peer-reviewed manuscripts published between January 2006 and May 2021. The 35 peer-reviewed articles included nine literature reviews delving into other themes while addressing barriers to help-seeking in their results and 26 clinical studies using quantitative (*n* = 14), qualitative (*n* = 9), or mixed methods (*n* = 3). The majority of the studies were conducted in the United States and focused on a population of men who had experienced sexual assault in childhood, adulthood, or military settings. Most articles were published within the first half of the eligible timeframe (2006–2013). Table 2 presents the main relevant findings and recommendations. The literature predominantly documented barriers to help-seeking, while findings on preferences for psychological services and motivators to help-seeking were more limited. See Figure 2 for an overview of the main topics of the thematic content analysis.

**Table 2.** Main Relevant Findings According to the Objectives of the Scoping Review

References	Aim of the studies	Main relevant findings	Main recommendations
Alaggia & Millington, 2008	• Understand the processes of disclosure and the effects of childhood SA on male and female survivors.	• Masculine norms and myths about male SA are barriers to help-seeking.	• Clinicians should be attentive to the needs of male survivors of SA and provide a supportive and accepting attitude.
Allard et al., 2011	• Review the body of available MST research and identify knowledge gaps.	• Masculine norms represent a barrier to help-seeking; • Military culture limits the disclosure of SA.	• No recommendation.
Dorahy & Clearwater, 2012	• Better understand the experiences of adult men who experienced SA.	• Shame and fear of others’ reaction and of retaliation limit disclosure; • Masculine norms and myths about male SA are barriers to help-seeking.	• Clinicians should tailor interventions to the needs of male victims of SA by facilitating disclosure.
Elder et al., 2017	• Examine male veterans’ perceptions of the effects of MST.	• Masculine norms are a barrier to help-seeking. • Fear of others’ reaction is a barrier to disclosure. • Substance abuse is a motivator to help-seeking.	• Interventions should be tailored to the consequences of male SA and be based on problem-solving.
Fogler et al., 2008	• Synthesize the knowledge on the effects of SA while considering the unique effects of clergy-perpetrated SA.	• Masculine norms represent a barrier to help-seeking.	• Clinicians should adopt a supporting attitude.
Hooper & Warwick, 2006	• Review gender data in relation to the prevalence of child SA, its impacts, and the service experience of adult survivors.	• Masculine norms and myths about male SA are barriers to help-seeking.	• No recommendation.
Hovey et al., 2011	• Facilitate a collaborative process to develop practical knowledge to improve the healthcare experiences of survivors of childhood SA into adulthood.	• Fear of the reaction of health care professionals to disclosure (being perceived as an abuser) and fear of being perceived as weak hinders help-seeking.	• Explore with men their preference for the clinician’s gender. Clinicians should adopt a supportive attitude.
Hoyt et al., 2011	• Review the prevalence and incidence rates of MST in men; • Discuss barriers to disclosure of male MST that may affect reported rates.	• Masculine norms are barriers to help-seeking. • Some values promoted in the military culture prevent men from disclosing MST.	• Interventions for MST should be tailored to veterans’ need.
Hoyt et al., 2012	• Present a model developed by Veterans Affairs for the conceptualization and treatment of men’s MST and PTSD and discuss considerations for its application in group therapy.	• Hierarchy among military can prevent men from disclosing MST and sharing their story in treatment; • Masculine norms are a barrier to help-seeking.	• No recommendation.
Isely et al., 2008	• Give voice to men who were abused as children by the Catholic clergy.	• Shame, guilt, anger, and fear of others’ reaction are barriers to disclosure of male SA.	• Clinicians should adopt a supportive attitude and establish a safe relationship with men.
Kakhnovets & Holohan, 2007	• Review the available scientific literature on the subject of male SA.	• Recommendation for interventions tailored to men’s need (i.e., educational component which deconstruct rape myths and masculine norms).	• No recommendation.
A. J. Khan et al., 2020	• Examine the impact of gender and MST on the effectiveness of TF-CBT in veterans in real-world clinical settings.	• Prolonged exposure, being action-oriented, may be more convenient for veterans who experienced MST.	• No recommendation.
Kia-Keating et al., 2010	• Better understand the experiences of male childhood SA survivors in connection with relationships.	• Male survivors of SA face relational challenges (e.g., intimacy, emotional expression) that pose barriers to seeking and maintaining relationships.	• Tailor interventions to male SA (e.g., target interpersonal strategies).
Light & Monk-Turner, 2009	• Explore how well the circumstances surrounding the SA and rape of men holds for a nationally representative sample.	• Men are more likely to seek help when they are physically injured during the SA and if they can pay for it.	• No recommendation.
Monk-Turner & Light, 2010	• Better understand what key factors shaped the likelihood of help-seeking among adult men who experienced SA.	• Men who experienced penetrative SA are less likely to seek help.	• Develop treatment models targeting the needs of men who experienced SA.
Morris et al., 2014	• Provide a review of available information on male veterans who experienced MST.	• Masculine norms and myths about male SA are barriers to help-seeking; • Military culture generates unique barriers to disclosing SA (e.g., importance of unit cohesion).	• No recommendation.
O’Brien et al., 2008	• Examine the role of alexithymia in the association with persistent trauma symptoms.	• Difficulty identifying feelings is related to the persistence of trauma symptoms.	• Interventions should be tailored to male survivors’ needs.
O’Brien et al., 2015	• Not reported.	• Masculine norms and rape myths constitute barriers further enhanced in the military; • Shame and guilt are barriers to disclosure that often result in incomplete trauma narrative; • MST-specific treatment options lack for men.	• Interventions should address myths and associated beliefs regarding male SA.
O’Leary & Gould, 2009	• Test if men who experienced childhood SA are overrepresented in suicidal ideation and if they significantly differ in their suicide attempts based on their clinical diagnosis; • Identify characteristics of abuse and reactions following assault that may increase suicidality.	• When assessing a problem, clinicians should explore the history of SA and its possible impact on the problem.	• No recommendation.
O’Leary, 2009	• Test if men’s social support and positive cognitive reframing are associated with better functioning, while avoidance and disengagement are associated with negative functioning.	• Professionals should be aware of male SA to offer a positive response when a man discloses, which could affect his psychological outcomes.	• No recommendation.
Pavao et al., 2013	• To estimate the proportion of homeless male and female veterans with MST and examine the link with mental health; • Describe the use of mental health and MST-related services among homeless women and men.	• Good access to mental health services is essential for the health of homeless veterans who experienced (or not) MST.	• No recommendation.
Peterson et al., 2011	• Systematically review the empirical literature on SA in men to assess the veracity of myths.	• Myths regarding male SA prevent men from discussing SA and from seeking help.	• No recommendation.
Sable et al., 2006	• Build knowledge about the perceived importance of barriers to reporting SA and rape, and explore gender differences within a group of students.	More men than women report: • Shame, guilt, confidentiality concerns, and fear of not being believed in regard to disclosure; • Unawareness about psychological services; • Distrust of the police and legal system.	• No recommendation.
Schry et al., 2015	• Provide preliminary data describing functional correlates of MST among male veterans to identify potential health care needs for this population.	• In case of MST history, clinicians should monitor symptoms severity, mental health condition, and suicidality.	• No recommendation.
Snipes et al., 2017	• Examine the theoretical influence of explicit power-sex beliefs on PTSD symptoms in college students.	• Treatment for men who experienced SA should examine power-sex beliefs (masculine norms).	• No recommendation.
Sorsoli et al., 2008	• Expand the current literature by exploring the nuances of men’s experiences of childhood SA.	• Rape myths, shame, fear of others’ reaction, and cultural norms impede disclosure and help-seeking; • Many men preferred a female clinician; • Clinicians must ask men unashamedly if they experienced SA.	• No recommendation.
Steiger et al., 2010	• Estimating the incidence and prevalence of SA in the Air Force ranks.	• Many barriers to help-seeking are reported: ignorance that what was experienced is a SA; lack of faith in the reporting process; fear of retaliation; minimization of the SA; confidentiality concerns; • Barriers related to military culture are reported.	• No recommendation.
Teram et al., 2006	• Better understand the experiences of male survivors of SA, particularly with respect to their encounters with health care professionals.	• Many men preferred female clinicians; • Masculine norms and myths regarding male SA represent a barrier to discussing childhood SA.	• Clinicians should adopt male-centric communication and emphasize a safe environment.
Turchik & Edwards, 2012	• To examine male rape myths and their origins in medicine, law, media, military, and prison settings; • Identify ways to eradicate male rape myths at individual, institutional, and societal levels.	• Male rape myths are embedded within society; • Military culture poses barriers to disclosure and help-seeking.	• Interventions should be tailored to men’s needs (e.g., sexuality, male rape myths).
Turchik et al., 2016	• Examine the need for a gender-sensitive conceptualization of SA and discuss the compatibility of current theories.	• Stigma and myths about male SA represent a barrier to discussing SA and seeking help.	• Interventions should address rape myths and masculinity.
Turchik et al., 2013	• Main objective: Better understand perceived barriers to accessing MST-related care for male veterans; • Secondary objective: Specifically explore men’s preferences regarding the gender of health care providers offering MST-related services.	• Men report stigma-related barriers to help-seeking, such as shame, confidentiality, self-blame, fear to not be believed, sensitivity, and reaction of provider; • Men report gender-related barriers to seeking help that target masculine norms; • Men report financial concerns and lack of knowledge about services; • Half of the men preferred a female clinician, a quarter preferred a male clinician, and others were indifferent.	• No recommendation.
Turchik et al., 2012	• Examine the proportion of veterans with a positive MST screen who receive MST-related care within 1 year; • Examine sociodemographic and military factors related to the use and the intensity of an MST care, and describe this care.	• Gender was the most consistent factor that affected both the use of MST-related care and intensity. Men were less likely to use MST-related care compared to women.	• Inclusive material, measures, and interview questions should be gender-specific to male MST.
Turchik et al., 2014	• Collect qualitative data from male veterans who experienced MST to design a gender-targeted MST brochure; • Quantitatively compare men’s evaluations of a neutral versus a gender-targeted psychoeducational brochure on MST; • Examine the effects of a pilot psychoeducational mail intervention on mental health and MST care utilization.	• Male veterans in the study preferred gender-specific material informing about MST.	• No recommendation.
Turchik, 2012	• Build on existing research to examine the relationship between college men’s sexual victim status and their engagement in health risk behaviors and sexual functioning.	• Clinicians’ awareness of unique issues related to male SA may foster seeking and continuing services for men who experienced MST; • Masculine norms and rape myths regarding male SA represent a barrier to discussing childhood SA.	• No recommendation.
Voelkel et al., 2015	• Examine the differences between men and women with and without MST.	• More women than men were linked to a service.	• No recommendation.
Voller et al., 2015	• Evaluate the utility of a conceptual model (associations among self-efficacy, male rape myths acceptance, devaluation of emotions, and psychiatric symptom severity) among male veterans who applied for PTSD disability benefits.	• Men devaluate emotions (masculine norms), which is associated with greater psychiatric symptoms and should be addressed during the treatment for male SA.	• No recommendation.
Wolfe et al., 2006	• Describe the long-term impact of physical and sexual abuse of boys by someone in a trusted, non-family relationship.	• The sensitivity and awareness regarding male SA and cultural, ethnic, and religious beliefs lack.	• No recommendation.

*Note.* MST = military sexual trauma; SA = sexual assault.

### Preferences Regarding Psychological Services

Five studies identified preferences regarding psychological services. These articles documented that men who experienced sexual assault show preferences pertaining both to the clinician and the psychological service (see Table 2).

#### Preferences Related to Clinician Characteristics

Three qualitative studies (Sorsoli et al., 2008; Teram et al., 2006; Turchik et al., 2013) reported that men who experienced sexual assault may express a preference for the gender of the clinician who will provide them with services. Some men may prefer to be assisted by a male clinician because they perceive that issues related to masculinity may be better understood or to avoid being vulnerable in front of a woman (Turchik et al., 2013). Other men appeared to find it easier to share sensitive information to a female clinician, whom they perceived as compassionate and less likely to have homophobic reactions (Sorsoli et al., 2008; Teram et al., 2006; Turchik et al., 2013).

In addition, four articles (three qualitative studies and one literature review) made specific recommendations to clinicians to adopt a supportive and accepting attitude when working with men who experienced sexual assault to enhance their sense of being understood and their receptivity to psychological help (Alaggia & Millington, 2008; Fogler et al., 2008; Hovey et al., 2011; Isely et al., 2008).

#### Preferences Related to the Type of Intervention

One retrospective cohort study (A. J. Khan et al., 2020) demonstrated that PE was more effective than CPT among men who experienced sexual assault in a military setting. Thus, the authors suggested that PE may be more suitable for male victims of military sexual assault due to its action-oriented and concrete nature.

In terms of intervention content, a randomized controlled trial (Turchik et al., 2014) reported that men who experienced sexual assault in a military context preferred to receive information about male-specific consequences to sexual assault than general information about sexual assault. Several studies (e.g., Elder et al., 2017; Kia-Keating et al., 2010; Monk-Turner & Light, 2010) extended these findings with recommendations (in discussion or in implications for practice) for developing and providing interventions that target the specific consequences and needs of men who have been sexually assaulted.

### Barriers to Help-Seeking

Several of the reviewed studies reported findings and recommendations regarding barriers to help-seeking in men who experienced sexual assault. Barriers fell into five categories, that is, related to male gender, to negative emotions (shame, blame, and guilt), to disclosure of sexual assault, to military context, and to accessibility of services (see Figure 2).

#### Barriers Related to Male Gender

The barriers to help-seeking most strongly supported by the reviewed literature were those related to male gender. A total of 14 studies addressing male sexual assault experienced in a variety of contexts (childhood, adulthood, and military service) identified adherence to traditional masculine norms as a barrier to help-seeking. Six of the studies were literature reviews, five were qualitative studies, one was a treatment study, and two were cross-sectional studies. The findings revealed that masculine identity may be an important issue to men who experienced sexual assault, who may feel the need to reassert their strength (O’Brien et al., 2015), self-reliance (e.g., Fogler et al., 2008; Hooper & Warwick, 2006), or emotional toughness (e.g., Hoyt et al., 2011; Turchik, 2012), in accordance with traditional masculine norms. Disclosing the assault and seeking help could therefore be viewed as signs of weakness (e.g., Hovey et al., 2011; Hoyt et al., 2011; Turchik, 2012).

The majority of studies that addressed masculine norms also identified myths about sexual assault (e.g., men cannot be sexually assaulted, men who are sexually assaulted are homosexual) as a barrier to disclosing sexual assault and to help-seeking. These myths were addressed in 12 studies (six literature reviews, four qualitative studies, and two cross-sectional studies). Men who experienced sexual assault reported fearing being labeled with unfounded or blunt stereotypes, such as they must have desired the assault, they are homosexual, they are not able to defend themselves, etc. (e.g., O’Brien et al., 2015; Teram et al., 2006; Turchik & Edwards, 2012). According to Turchik and Edwards’ (2012) literature review, these myths about sexual assault could invalidate the experience of sexually assaulted men and contradict their masculine identity. Several authors have therefore recommended integrating an educational component into interventions to deconstruct the impact of traditional masculine norms and myths about male sexual assault (e.g., Kakhnovets & Holohan, 2007; O’Brien et al., 2015).

#### Shame, Blame, and Guilt

Shame, self-blame, and guilt themselves act as a barrier to help-seeking. Six studies (two literature reviews, two qualitative studies, and two cross-sectional studies) have identified that feelings of shame, guilt, or self-blame for causing or failing to prevent the sexual assault were a barrier to disclosing sexual assault. The qualitative studies of Dorahy and Clearwater (2012) and of Isely and colleagues (2008) illustrated that overwhelming shame and guilt following childhood sexual abuse led to a number of other obstacles hampering help-seeking, such as a sense of inferiority and incompetence, low self-esteem, excessive anger or rage, and denial. Furthermore, O’Brien and colleagues’ (2015) literature review documented that men who did seek help often offered an incomplete trauma narrative (e.g., physical reaction) at the beginning of treatment, due to shame and guilt.

#### Disclosure

Ten studies identified various considerations about disclosing sexual assault to receive help as a barrier to help-seeking. Seven studies (e.g., Dorahy & Clearwater, 2012; Elder et al., 2017; Turchik et al., 2013) noted that men could fear or anticipate negative reaction (e.g., anger, laughter, denial) from relatives and health service providers on disclosure of the sexual assault, often related to myths about male sexual assault. One qualitative study (Dorahy & Clearwater, 2012) and one research report (Steiger et al., 2010) described a fear of retaliation (e.g., loss of relationships or social status, punishment for an unperformed violation) upon disclosure. Moreover, three studies identified confidentiality concerns among male victims of sexual assault when disclosing and seeking psychological help (Sable et al., 2006; Steiger et al., 2010; Turchik et al., 2013), and two studies highlighted issues of distrust and negative experiences with the justice system among this population (Sable et al., 2006; Steiger et al., 2010).

#### Military Culture

Five peer-reviewed articles (four literature reviews and one treatment study) and a research report (Steiger et al., 2010) identified that military culture generates unique barriers to disclosure for veterans who are victims of sexual assault, or exacerbates other existing barriers (e.g., myths about male sexual assault). The reviewed studies documented that men who are sexually assaulted in military service may be compelled to remain silent. They may feel the need to conform to the values and rules of military culture (e.g., unit cohesion) or fear that disclosing an assault by a fellow service member will be perceived as a betrayal and lead to negative consequences (e.g., Morris et al., 2014; O’Brien et al., 2015; Steiger et al., 2010).

#### Accessibility of Services

Perceived and actual accessibility of services was another barrier to help-seeking. Three studies (O’Brien et al., 2015; Pavao et al., 2013; Voelkel et al., 2015) indicated that more female than male victims of military sexual trauma received psychological services, notably because greater resources were allocated to women in specialized sexual assault services. Other barriers (i.e., financial concerns, lack of knowledge about available services, cultural barriers, lack of sensitivity, and awareness regarding religious beliefs) were also identified across four other studies (e.g., Light & Monk-Turner, 2009; Sorsoli et al., 2008; Turchik et al., 2013; Wolfe et al., 2006).

### Motivators to Help-Seeking

The reviewed studies reported motivators to help-seeking for men who experienced sexual assault, documented in one cross-sectional study (Light & Monk-Turner, 2009) and one qualitative study (Elder et al., 2017). Both studies identified that male victims of sexual assault may be more likely to seek services for a co-occurring problem (see Figure 2). Light and Monk-Turner (2009) suggested that physical injuries resulting from the sexual assault could legitimize help-seeking for some men. In addition, Elder et al.’s (2017) study revealed that male victims of sexual assault who use alcohol or drugs excessively may be more inclined to seek help when their substance use reaches a severity requiring medical or addiction services.

## Discussion

The objective of this scoping review was to document preferences of men who experienced sexual assault regarding psychological services, and to explore barriers and motivators to help-seeking. To optimize TF-CBT, researchers and clinicians could accommodate preferences regarding psychological services, alleviate barriers to help-seeking, and consider motivators to help-seeking for men who experienced sexual assault. This scoping review could contribute to guide adaptation of TF-CBT to men who experienced sexual assault.

The gender of the clinician was the preference that was most documented in the literature, although there was no consensus toward one specific gender (e.g., Turchik et al., 2013). Female clinicians were perceived as more compassionate and less likely to have homophobic reactions while male clinicians were perceived as more understanding of masculine issues (Sorsoli et al., 2008; Teram et al., 2006; Turchik et al., 2013). Thus, the opportunity to select the gender of the clinician should be given to men who experienced sexual assault, whenever possible. In addition, preferences leaned toward active and sensitive interventions tailored to male sexual assault and its consequences (A. J. Khan et al., 2020; Turchik et al., 2014). As PE is a more concrete and action-oriented, problem-solving approach than other therapeutic options (such as CPT), it may respond more adequately to the needs of men who experienced sexual assault.

Nearly half of the reviewed articles identified masculine norms and myths about male sexual assault as barriers to help-seeking, underlining the need to address these. Clinicians and other professionals should be educated about masculine norms and myths about male sexual assault. Indeed, it would enable them to adapt TF-CBT to male victims of sexual assault by adding an important psychoeducational component to deconstruct these myths and norms, as recommended by several studies in this scoping review (e.g., Kakhnovets & Holohan, 2007; O’Brien et al., 2015). This adaptation requires special attention when TF-CBT is offered in the military population since the values and rules of military culture create and reinforce traditional masculine norms (e.g., strength) and myths about sexual assault (such as the belief that men cannot be victim of the sexual assault; e.g., Morris et al., 2014; O’Brien et al., 2015).

This scoping review identified that shame, self-blame, and guilt can inhibit disclosure of sexual assault among men, hindering help-seeking behavior and often leading to the concealment of crucial details during trauma narrative (e.g., Dorahy & Clearwater, 2012; O’Brien et al., 2015). Considering this particular need, one way to adapt TF-CBT to male sexual assault victims is to proactively validate sexual assault experience and normalize the emotions and consequences associated with male sexual assault. This means that the therapist will also need to show extra sensitivity when enquiring about the assault and to avoid forcing disclosure or putting men in an unnecessary vulnerability position that may exacerbate negative emotions (e.g., shame for not defending themselves) and confront masculine identity.

Some studies documented the limited accessibility of psychological services for men who experienced sexual assault (e.g., O’Brien et al., 2015; Voelkel et al., 2015). One way to alleviate this barrier could be to develop online TF-CBT dedicated specifically to men, as it may allow men who experienced sexual assault to self-manage their difficulties, to protect their confidentiality, to reduce the risk of stigmatization, and to facilitate access to evidence-based strategies to alleviate symptoms. Randomized controlled trials have demonstrated that online TF-CBT can be effective in reducing PTSD, depression, anxiety, and insomnia symptoms (e.g., Belleville et al., 2023; Littleton et al., 2016; Spence et al., 2011).

The reviewed studies offered avenues on what motivates men who experienced sexual assault to seek help. Men appear more likely to seek help in the presence of a co-occurring problem (substance abuse, severe physical injury; Elder et al., 2017; Light & Monk-Turner, 2009). Thus, history of sexual assault could be systematically assessed in a non-confronting way in medical (e.g., hospitals, private medical clinics) or addiction settings (e.g., rehabilitation centers, men’s support organizations). Moreover, specialized sexual assault services such as TF-CBT could be promoted within these services for reference purposes.

### Limitations

Although this scoping review provided useful insights about preferences regarding psychological services, as well as barriers and motivators to help-seeking for men who experienced sexual assault, the results should be interpreted in light of some limitations. First, the search strategy was based on uncontrolled vocabulary and restricted to three databases. Therefore, some studies may have been missed. However, the method was standardized and based on the best practice guidelines for conducting scoping reviews (Munn et al., 2022; Tricco et al., 2018), and the use of 23 data equations across three databases from the disciplines of medicine (PubMed), psychology (PsycNet), and education (Eric) enabled a thorough mapping of literature. Furthermore, the quality of the included studies was not assessed, which even if in line with the best practice guidelines for scoping reviews (Tricco et al., 2018), limits confidence about the robustness of the reported findings. Another caveat comes from the limited availability of empirical data on the preferences regarding psychological services from men who experienced sexual assault. Although several authors offered recommendations on this topic, as documented in Table 2, the lack of supportive evidence underscored the need for further empirical investigation. Including barriers and motivators as variables of interest may offer additional evidence-based recommendations to further adapt TF-CBT. Despite these limitations, this scoping review was the first to have a primary aim of exploring preferences regarding psychological services among men who experienced sexual assault. It provided rich results on different components of help-seeking (preferences, barriers, and motivators). The use of a thematic content analysis helped to organize the available literature to offer pragmatic ideas to target the necessary adaptations to TF-CBT for men who experienced sexual assault.

### Conclusion

In conclusion, men who experienced sexual assault have preferences toward psychological services but also face barriers to seeking psychological help. Despite a substantial body of peer-reviewed literature documented barriers to help-seeking, empirical data on preferences regarding psychological services are scarce, indicating a notable knowledge gap. Moreover, existing literature predominantly focuses on very specific preferences regarding psychological services, often using qualitative methodologies, and primarily among men who have been sexually assaulted in the military. To address this gap, further research is needed to comprehensively document preferences regarding psychological services across various clinical and demographic contexts. Potential research avenues could involve distinguishing preferences for psychological services among men who experienced sexual assault according to the context of the assault (e.g., childhood, adulthood, in the military) and according to identity factors (e.g., ethnicity, ability status, sexual orientation). In light of findings of this scoping review regarding PE, further treatment studies focusing on this therapy are necessary to demonstrate whether it aligns with the preferences of men who experienced sexual assault, and whether this alignment enhances treatment response. This research could provide valuable guidance for clinicians and researchers in adapting TF-CBT treatment plans for men who experienced sexual assault. Such studies could provide further insight on how to mobilize men toward psychological services and to maximize the efficacy of TF-CBT according to their preferences and needs.

## Appendix

### Search Equations

1. “Men” AND “PTSD” AND “cognitive behavioral therapy”
2. “Men” AND “sexual assault” AND “PTSD” AND “cognitive behavioral therapy”
3. “Men” AND “PTSD” AND “cognitive behavioral therapy” AND “differences”
4. “PTSD” AND “cognitive behavioral therapy” AND “efficacy”
5. “Gender differences” AND “PTSD” AND “cognitive behavioral therapy”
6. “Sex differences” AND “PTSD” AND “cognitive behavioral therapy”
7. “Sex differences” AND “PTSD” AND “cognitive behavioral treatment”
8. “Men” AND “PTSD” AND “treatment”
9. “Men” AND “PTSD” AND “treatment” AND “sexual assault”
10. “Men” AND “PTSD” AND “treatment” AND “sexual abuse”
11. “Men” AND “sexual abuse” AND “PTSD” AND “experiences”
12. “Men” AND “sexual assault” AND “PTSD” AND “experiences”
13. “Men” AND “women” AND “differences” AND “PTSD”
14. “Men” AND “women” AND “differences” AND “PTSD symptoms”
15. “Men” AND “women” AND “differences” AND “PTSD” AND “treatment”
16. “Men” AND “women” AND “differences” AND “PTSD” AND “treatment” AND “cognitive behavioral”
17. “PTSD” AND “treatment” AND “gender preferences”
18. “PTSD” AND “treatment” AND “gender preferences” AND “men”
19. “PTSD” AND “treatment” AND “gender preferences” AND “male”
20. “PTSD” AND “treatment” AND “gender preferences” AND “sexual assault”
21. “PTSD” AND “treatment” AND “gender preferences” AND “sexual abuse”
22. “PTSD” AND “intervention” AND “gender preferences”
23. “PTSD” AND “intervention” AND “gender preferences” AND “sexual assault”

## References

[bibr1-15579883241260512] Alaggia R. Millington G. (2008). Male child sexual abuse: A phenomenology of betrayal. Clinical Social Work Journal, 36, 265–275. https://doi.org/c35mnx

[bibr2-15579883241260512] AL-Asadi A. M. (2021). Comparison between male and female survivors of sexual abuse and assault in relation to age at admission to therapy, age of onset, and age at last sexual assault: Retrospective observational study. JMIRx Med, 2(4), Article e23713. https://doi.org/kqw8 10.2196/23713 PMC10414400 37725544

[bibr3-15579883241260512] Allard C. B. Nunnink S. Gregory A. M. Klest B. Platt M. (2011). Military sexual trauma research: A proposed agenda. Journal of Trauma & Dissociation, 12(3), 324–345. https://doi.org/bfcqww 21534099 10.1080/15299732.2011.542609

[bibr4-15579883241260512] Békés V. Beaulieu-Prévost D. Guay S. Belleville G. Marchand A. (2016). Women with PTSD benefit more from psychotherapy than men. Psychological Trauma: Theory, Research, Practice, and Policy, 8(6), 720–727. 10.1037/tra0000122 27031079

[bibr5-15579883241260512] Belleville G. Ouellet M. C. Békés V. Lebel J. Morin C. M. Bouchard S. Guay S. Bergeron N. Ghosh S. Campbell T. Macmaster F. P. (2023). Efficacy of a therapist-assisted self-help internet-based intervention targeting PTSD, depression, and insomnia symptoms after a disaster: A randomized controlled trial. Behavior Therapy, 54(2), 230–246. https://doi.org/kqxc 36858756 10.1016/j.beth.2022.08.004

[bibr6-15579883241260512] Bisson J. Roberts N. P. Andrew M. Cooper R. Lewis C. (2013). Psychological therapies for chronic post-traumatic stress disorder (PTSD) in adults. Cochrane Database of Systematic Reviews, 12, Article CD003388. https://doi.org/gd24xx 10.1002/14651858.CD003388.pub4 PMC6991463 24338345

[bibr7-15579883241260512] Chard K. M. (2005). An evaluation of cognitive processing therapy for the treatment of posttraumatic stress disorder related to childhood sexual abuse. Journal of Consulting and Clinical Psychology, 73(5), 965–971. https://doi.org/cn7x3p 16287396 10.1037/0022-006X.73.5.965

[bibr8-15579883241260512] Covers M. L. V. de Jongh A. Huntjens R. J. C. de Roos C. van den Hout M. Bicanic I. A. E. (2021). Early intervention with eye movement desensitization and reprocessing (EMDR) therapy to reduce the severity of post-traumatic stress symptoms in recent rape victims : A randomized controlled trial. European Journal of Psychotraumatology, 12(1), 1–16. 10.1080/20008198.2021.1943188 34531963 PMC8439210

[bibr9-15579883241260512] Cusack K. Jonas D. E. Forneris C. A. Wines C. Sonis J. Middleton J. C. Feltner C. Brownley K. A. Olmsted K. R. Greenblatt A. Weil A. Gaynes B. N. (2016). Psychological treatments for adults with posttraumatic stress disorder: A systematic review and meta-analysis. Clinical Psychology Review, 43, 128–141. https://doi.org/gdcgqs 26574151 10.1016/j.cpr.2015.10.003

[bibr10-15579883241260512] Dorahy M. J. Clearwater K. (2012). Shame and guilt in men exposed to childhood sexual abuse: A qualitative investigation. Journal of Child Sexual Abuse, 21(2), 155–175. https://doi.org/gjm7z3 22452299 10.1080/10538712.2012.659803

[bibr11-15579883241260512] Ehring T. Welboren R. Morina N. Wicherts J. M. Freitag J. Emmelkamp P. M. G. (2014). Meta-analysis of psychological treatments for posttraumatic stress disorder in adult survivors of childhood abuse. Clinical Psychology Review, 34(8), 645–657. https://doi.org/f6s4v2 25455628 10.1016/j.cpr.2014.10.004

[bibr12-15579883241260512] Elder W. B. Domino J. L. Mata-Galán E. L. Kilmartin C. (2017). Masculinity as an avoidance symptom of posttraumatic stress. Psychology of Men & Masculinity, 18(3), 198–207. https://doi.org/gcgs8k

[bibr13-15579883241260512] Elliott D. M. Mok D. S. Briere J. (2004). Adult sexual assault : Prevalence, symptomatology, and sex differences in the general population. Journal of Traumatic Stress, 17(3), 203–211. https://doi.org/cb35b5 15253092 10.1023/B:JOTS.0000029263.11104.23

[bibr14-15579883241260512] Felmingham K. L. Bryant R. A. (2012). Gender differences in the maintenance of response to cognitive behavior therapy for posttraumatic stress disorder. Journal of Consulting and Clinical Psychology, 80(2), 196–200. https://doi.org/f3wrds 22309472 10.1037/a0027156

[bibr15-15579883241260512] Fogler J. M. Shipherd J. C. Clarke S. Jensen J. Rowe E. (2008). The impact of clergy-perpetrated sexual abuse: The role of gender, development, and posttraumatic stress. Journal of Child Sexual Abuse, 17(3–4), 329–358. https://doi.org/dpqnxj 19042605 10.1080/10538710802329940

[bibr16-15579883241260512] Forman-Hoffman V. Middleton J. C. Feltner C. Gaynes B. N. Weber R. P. Bann C. Viswanathan M. Lohr K. N. Baker C. Green J. (2018). Psychological and pharmacological treatments for adults with posttraumatic stress disorder: A systematic review update. Agency for Healthcare Research and Quality. https://www.ncbi.nlm.nih.gov/books/NBK525132/30204376

[bibr17-15579883241260512] Gallegos A. M. Wolff K. B. Streltzov N. A. Adams L. B. Carpenter-Song E. Nicholson J. Stecker T. (2015). Gender differences in service utilization among OEF/OIF veterans with posttraumatic stress disorder after a brief cognitive behavioral intervention to increase treatment engagement: A mixed methods study. Women’s Health Issues, 25(5), 542–547. https://doi.org/ghvc3r 26051022 10.1016/j.whi.2015.04.008 PMC4569511

[bibr18-15579883241260512] Guina J. Nahhas R. W. Kawalec K. Farnsworth S. (2019). Are gender differences in DSM-5 PTSD symptomatology explained by sexual trauma? Journal of Interpersonal Violence, 34(21–22), 4713–4740. https://doi.org/gg6fdn 27827321 10.1177/0886260516677290

[bibr19-15579883241260512] Heidt J. M. Marx B. P. Forsyth J. P. (2005). Tonic immobility and childhood sexual abuse : A preliminary report evaluating the sequela of rape induced paralysis. Behaviour Research and Therapy, 43(9), 1157–1171. https://doi.org/ff9g23 16005703 10.1016/j.brat.2004.08.005

[bibr20-15579883241260512] Hooper C.-A. Warwick I. (2006). Gender and the politics of service provision for adults with a history of childhood sexual abuse. Critical Social Policy, 26(2), 467–479. https://doi.org/fcpkcs

[bibr21-15579883241260512] Hovey A. Stalker C. A. Schachter C. L. Teram E. Lasiuk G. (2011). Practical ways psychotherapy can support physical healthcare experiences for male survivors of childhood sexual abuse. Journal of Child Sexual Abuse, 20(1), 37–57. https://doi.org/cqwtm8 21259146 10.1080/10538712.2011.539963

[bibr22-15579883241260512] Hoyt T. Klosterman Rielage J. Williams L. F. (2011). Military sexual trauma in men: A review of reported rates. Journal of Trauma & Dissociation, 12(3), 244–260. https://doi.org/fmxz9z 21534094 10.1080/15299732.2011.542612

[bibr23-15579883241260512] Hoyt T. Rielage J. K. Williams L. F. (2012). Military sexual trauma in men: Exploring treatment principles. Traumatology, 18(3), 29–40. https://doi.org/fmxz9z

[bibr24-15579883241260512] Isely P. J. Isely P. Freiburger J. McMackin R. (2008). In their own voices: A qualitative study of men abused as children by Catholic clergy. Journal of Child Sexual Abuse, 17(3–4), 201–215. https://doi.org/fmj5ph 19042598 10.1080/10538710802329668

[bibr25-15579883241260512] Kakhnovets R. Holohan D. R. (2007). Initial steps in treating the male patient. Federal Practitioner, 24, 16–29.

[bibr26-15579883241260512] Khan A. J. Holder N. Li Y. Shiner B. Madden E. Seal K. Neylan T. C. Maguen S. (2020). How do gender and military sexual trauma impact PTSD symptoms in cognitive processing therapy and prolonged exposure? Journal of Psychiatric Research, 130, 89–96. https://doi.org/gm5jt3 32798774 10.1016/j.jpsychires.2020.06.025

[bibr27-15579883241260512] Khan S. Greene J. Mellins C. A. Hirsch J. S. (2020). The social organization of sexual assault. Annual Review of Criminology, 3, 139–163.

[bibr28-15579883241260512] Kia-Keating M. Sorsoli L. Grossman F. K. (2010). Relational challenges and recovery processes in male survivors of childhood sexual abuse. Journal of Interpersonal Violence, 25(4), 666–683. https://doi.org/gm5jt3 19465573 10.1177/0886260509334411

[bibr29-15579883241260512] Light D. Monk-Turner E. (2009). Circumstances surrounding male sexual assault and rape: Findings from the National Violence Against Women Survey. Journal of Interpersonal Violence, 24(11), 1849–1858. https://doi.org/gm5jt3 18981191 10.1177/0886260508325488

[bibr30-15579883241260512] Littleton H. Grills A. E. Kline K. D. Schoemann A. M. Dodd J. C. (2016). The from survivor to thriver program: RCT of an online therapist-facilitated program for rape-related PTSD. Journal of Anxiety Disorders, 43, 41–51. https://doi.org/f88j3j 27513363 10.1016/j.janxdis.2016.07.010 PMC5056149

[bibr31-15579883241260512] Masho S. W. Alvanzo A. (2010). Help-seeking behaviors of men sexual assault survivors. American Journal of Men’s Health, 4(3), 237–242. https://doi.org/fr7tmj 10.1177/1557988309336365 19706673

[bibr32-15579883241260512] Monk-Turner E. Light D. (2010). Male sexual assault and rape: Who seeks counseling. Sexual Abuse: A Journal of Research and Treatment, 22(3), 255–265. https://doi.org/fk7839 20713746 10.1177/1079063210366271

[bibr33-15579883241260512] Morris E. E. Smith J. C. Farooqui S. Y. Surís A. M. (2014). Unseen battles : The recognition, assessment, and treatment issues of men with military sexual trauma (MST). Trauma, Violence, & Abuse, 15(2), 94–101. https://doi.org/gf4rn6 10.1177/1524838013511540 24231941

[bibr34-15579883241260512] Munn Z. Pollock D. Khalil H. Alexander L. Mclnerney P. Godfrey C. M. Peters M. Tricco A. C. (2022). What are scoping reviews? Providing a formal definition of scoping reviews as a type of evidence synthesis. JBI Evidence Synthesis, 20(4), 950–952. https://doi.org/js3z 35249995 10.11124/JBIES-21-00483

[bibr35-15579883241260512] O’Brien C. Gaher R. M. Pope C. Smiley P. (2008). Difficulty identifying feelings predicts the persistence of trauma symptoms in a sample of veterans who experienced military sexual trauma. The Journal of Nervous and Mental Disease, 196(3), 252–255. https://doi.org/drps4k 18340263 10.1097/NMD.0b013e318166397d

[bibr36-15579883241260512] O’Brien C. Keith J. Shoemaker L. (2015). Don’t tell: Military culture and male rape. Psychological Services, 12(4), 357–365. https://doi.org/f7zmjc 26524277 10.1037/ser0000049

[bibr37-15579883241260512] O’Leary P. J. (2009). Men who were sexually abused in childhood: Coping strategies and comparisons in psychological functioning. Child Abuse & Neglect, 33(7), 471–479. https://doi.org/d7h4gw 19589595 10.1016/j.chiabu.2009.02.004

[bibr38-15579883241260512] O’Leary P. J. Gould N. (2009). Men who were sexually abused in childhood and subsequent suicidal ideation: Community comparison, explanations and practice implications. British Journal of Social Work, 39(5), 950–968. https://doi.org/dmz7fh

[bibr39-15579883241260512] Pavao J. Turchik J. A. Hyun J. K. Karpenko J. Saweikis M. McCutcheon S. Kane V. Kimerling R. (2013). Military sexual trauma among homeless veterans. Journal of General Internal Medicine, 28, 536–541. https://doi.org/gbdfsp 10.1007/s11606-013-2341-4 PMC3695264 23807062

[bibr40-15579883241260512] Peterson Z. D. Voller E. K. Polusny M. A. Murdoch M. (2011). Prevalence and consequences of adult sexual assault of men: Review of empirical findings and state of the literature. Clinical Psychology Review, 31(1), 1–24. https://doi.org/b7jqhh 21130933 10.1016/j.cpr.2010.08.006

[bibr41-15579883241260512] Resick P. A. Schnicke M. K. (1992). Cognitive processing therapy for sexual assault victims. Journal of Consulting and Clinical Psychology, 60(5), 748–756. https://doi.org/ckkp7q 1401390 10.1037//0022-006x.60.5.748

[bibr42-15579883241260512] Sable M. R. Danis F. Mauzy D. L. Gallagher S. K. (2006). Barriers to reporting sexual assault for women and men: Perspectives of college students. Journal of American College Health, 55(3), 157–162. https://doi.org/bvtpnh 17175901 10.3200/JACH.55.3.157-162

[bibr43-15579883241260512] Schnurr P. P. Chard K. M. Ruzek J. I. Chow B. K. Resick P. A. Foa E. B. Marx B. P. Friedman M. J. Bovin M. J. Caudle K. L. Castillo D. Curry K. T. Hollifield M. Huang G. D. Chee C. L. Astin M. C. Dickstein B. Renner K. Clancy C. P. . . .Shih M.-C. (2022). Comparison of prolonged exposure vs cognitive processing therapy for treatment of posttraumatic stress disorder among US veterans: A randomized clinical trial. JAMA Network Open, 5(1), Article e2136921. https://doi.org/gn7kdd 10.1001/jamanetworkopen.2021.36921 PMC8771295 35044471

[bibr44-15579883241260512] Schnyder U. Cloitre M. (Eds.). (2022). Evidence based treatments for trauma-related psychological disorders: A clinical practice guide (2nd ed.). Springer. https://doi.org/kqw9

[bibr45-15579883241260512] Schry A. R. Hibberd R. Wagner H. R. Turchik J. A. Kimbrel N. A. Wong M. . . .Brancu M. (2015). Functional correlates of military sexual assault in male veterans. Psychological Services, 12(4), 384–393. https://doi.org/gf4rm6 26524280 10.1037/ser0000053

[bibr46-15579883241260512] Snipes D. J. Calton J. M. Green B. A. Perrin P. B. Benotsch E. G. (2017). Rape and posttraumatic stress disorder (PTSD): Examining the mediating role of explicit sex power beliefs for men versus women. Journal of Interpersonal Violence, 32(16), 2453–2470. https://doi.org/gbqhws 26141347 10.1177/0886260515592618

[bibr47-15579883241260512] Sorsoli L. Kia-Keating M. Grossman F. K. (2008). “I keep that hush-hush”: Male survivors of sexual abuse and the challenges of disclosure. Journal of Counseling Psychology, 55(3), 333–345. https://doi.org/fc5bzk

[bibr48-15579883241260512] Spence J. Titov N. Dear B. F. Johnston L. Solley K. Lorian C. Wootton B. Zou J. Schwenke G. (2011). Randomized controlled trial of internet-delivered cognitive behavioral therapy for posttraumatic stress disorder. Depression and Anxiety, 28(7), 541–550. 21721073 10.1002/da.20835

[bibr49-15579883241260512] Steiger D. M. Chattopadhyay M. Rao M. Green E. Nemeckay K. Yen E. (2010, December). Findings from the 2010 prevalence/incidence survey of sexual assault in the Air Force. Air Force Magazine, 1–98.

[bibr50-15579883241260512] Swift J. K. Callahan J. L. Cooper M. Parkin S. R. (2018). The impact of accommodating client preference in psychotherapy: A meta-analysis. Journal of Clinical Psychology, 74(11), 1924–1937. https://doi.org/gfnvzb 30091140 10.1002/jclp.22680

[bibr51-15579883241260512] Teram E. Stalker C. Hovey A. Schachter C. Lasiuk G. (2006). Towards male-centric communication: Sensitizing health professionals to the realities of male childhood sexual abuse survivors. Issues in Mental Health Nursing, 27(5), 499–517. https://doi.org/gfnvzb 16613801 10.1080/01612840600599994

[bibr52-15579883241260512] Tewksbury R. (2007). Physical, mental and sexual consequences. International Journal of Men’s Health, 6, 22–35.

[bibr53-15579883241260512] Tourigny M. Hébert M. Joly J. Cyr M. Baril K. (2008). Prevalence and co-occurrence of violence against children in the Quebec population. Australian and New Zealand Journal of Public Health, 32(4), 331–335. https://doi.org/c7974k 18782395 10.1111/j.1753-6405.2008.00250.x

[bibr54-15579883241260512] Tricco A. C. Lillie E. Zarin W. O’Brien K. K. Colquhoun H. Levac D. . . .Straus S. E. (2018). PRISMA extension for scoping reviews (PRISMAScR): Checklist and explanation. Annals of Internal Medicine, 169(7), 467–473. https://doi.org/gfd8vk 30178033 10.7326/M18-0850

[bibr55-15579883241260512] Turchik J. A. (2012). Sexual victimization among male college students: Assault severity, sexual functioning, and health risk behaviors. Psychology of Men & Masculinity, 13(3), 243–255. https://doi.org/gf4rqd

[bibr56-15579883241260512] Turchik J. A. Edwards K. M. (2012). Myths about male rape : A literature review. Psychology of Men & Masculinity, 13(2), 211–226. https://doi.org/bgpw9v

[bibr57-15579883241260512] Turchik J. A. Hebenstreit C. L. Judson S. S. (2016). An Examination of the gender inclusiveness of current theories of sexual violence in adulthood: Recognizing male victims, female perpetrators, and same sex violence. Trauma, Violence & Abuse, 17(2), 133–148. https://doi.org/gfnvzb 10.1177/1524838014566721 25612800

[bibr58-15579883241260512] Turchik J. A. McLean C. Rafie S. Hoyt T. Rosen C. S. Kimerling R. (2013). Perceived barriers to care and provider gender preferences among veteran men who have experienced military sexual trauma: A qualitative analysis. Psychological Services, 10(2), 213–222. https://doi.org/gf4rqd 22984877 10.1037/a0029959

[bibr59-15579883241260512] Turchik J. A. Pavao J. Hyun J. Mark H. Kimerling R. (2012). Utilization and intensity of outpatient care related to military sexual trauma for veterans from Afghanistan and Iraq. The Journal of Behavioral Health Services & Research, 39, 220–233. https://doi.org/gf4rqd 22396046 10.1007/s11414-012-9272-4

[bibr60-15579883241260512] Turchik J. A. Rafie S. Rosen C. S. Kimerling R. (2014). Preferences for gender targeted health information: A study of male veterans who have experienced military sexual trauma. American Journal of Men’s Health, 8(3), 240–248. https://doi.org/gf4rqd 10.1177/1557988313508304 24232582

[bibr61-15579883241260512] Voelkel E. Pukay-Martin N. D. Walter K. H. Chard K. M. (2015). Effectiveness of cognitive processing therapy for male and female US veterans with and without military sexual trauma. Journal of Traumatic Stress, 28(3), 174–182. https://doi.org/f7qrzf 25976767 10.1002/jts.22006

[bibr62-15579883241260512] Voller E. Polusny M. A. Noorbaloochi S. Street A. Grill J. Murdoch M. (2015). Self-efficacy, male rape myth acceptance, and devaluation of emotions in sexual trauma sequelae: Findings from a sample of male veterans. Psychological Services, 12(4), 420–427. https://doi.org/f7zr8v 26524284 10.1037/ser0000046

[bibr63-15579883241260512] Wolfe D. A. Francis K. J. Straatman A. L. (2006). Child abuse in religiously affiliated institutions: Long-term impact on men’s mental health. Child Abuse & Neglect, 30(2), 205–212. https://doi.org/dw62vx 16464495 10.1016/j.chiabu.2005.08.015

